# Cu Nanoparticles in Hydrogels of Chitosan-PVA Affects the Characteristics of Post-Harvest and Bioactive Compounds of Jalapeño Pepper

**DOI:** 10.3390/molecules22060926

**Published:** 2017-06-02

**Authors:** Zeus H. Pinedo-Guerrero, Alma Delia Hernández-Fuentes, Hortensia Ortega-Ortiz, Adalberto Benavides-Mendoza, Gregorio Cadenas-Pliego, Antonio Juárez-Maldonado

**Affiliations:** 1 Departamento de Horticultura, Universidad Autónoma Agraria Antonio Narro, 25315 Saltillo, Coahuila, Mexico; ing.zeuspinedo@gmail.com (Z.H.P.-G.); abenmen@gmail.com (A.B.-M.); 2 Instituto de Ciencias Agropecuarias, Universidad Autónoma del Estado de Hidalgo, Tulancingo, 43600 Hidalgo, Mexico; hfad@hotmail.com; 3 Centro de Investigación en Química Aplicada, 25294 Saltillo, Coahuila, Mexico; hortensia.ortega@ciqa.edu.mx (H.O.-O.); gregorio.cadenas@ciqa.edu.mx (G.C.-P.); 4 Departamento de Botánica, Universidad Autónoma Agraria Antonio Narro, 25315 Saltillo, Coahuila, Mexico

**Keywords:** hydrogels of chitosan-PVA, bioactive compounds, capsaicin, antioxidants, jalapeño pepper, post-harvest.

## Abstract

Peppers are consumed all over the world due to the flavor, aroma, and color that they add to food. Additionally, they play a role in human health, as they contain a high concentration of bioactive compounds and antioxidants. The treatments used were an absolute control, Cs-PVA, and four treatments with 0.02, 0.2, 2, and 10 mg (nCu) g^−1^ (Cs-PVA). The application of Cu nanoparticles in chitosan-PVA hydrogels increases the content of capsaicin by up to 51% compared to the control. This application also increases the content of antioxidants ABTS [2,2′-azino-bis (3-ethylbenzothiazolin-6-sulfonic acid)] and DPPH (2,2-diphenyl-1-picrylhydrazyl), total phenols and flavonoids (4%, 6.6%, 5.9%, and 12.7%, respectively) in jalapeño pepper fruits stored for 15 days at room temperature; under refrigeration, it increases DPPH antioxidants, total phenols, and flavonoids (23.9%, 1.54%, and 17.2%, respectively). The application of Cu nanoparticles in chitosan-PVA hydrogels, even when applied to the substrate, not only has an effect on the development of the jalapeño pepper crop, but also modifies the post-harvest characteristics of the jalapeño pepper fruits.

## 1. Introduction

Mexico is the second greatest producer of fresh peppers worldwide, with an area of 149,000 hectares, and its main export destinations are the United States, Canada, and Spain, among others [1]. In Mexico, peppers are the eighth most economically valuable crop, with an average production volume of 2.2 million tons, of which approximately 900 thousand tons of fresh, dry, and processed peppers are exported [2]; in 2016, the production of peppers in its different varieties reached 2.3 million tons [1].

Peppers are consumed all over the world due to the flavor, aroma, and color that they add to food. In addition to their sensory importance, peppers play a role in human health, as they contain a high concentration of bio-functional compounds and antioxidants that are important in the prevention of cardiovascular diseases, cancer, and neurological disorders. The main compounds are capsaicin and dihydrocapsaicin; these compounds are synthesized in fruits [3], are responsible for the pungency, and represent 80–90% of the capsaicinoids of pepper species [4]. Excess reactive oxygen species (ROS) are associated with various diseases in humans and have led to an increased consumption in this type of food that reduces oxidative damage in biological systems [5]. 

Nanotechnology has led to great expectations for the development of new products and applications in a wide range of industrial and human consumption sectors. It is expected that this technology will revolutionize the entire food chain, ranging from production to processing and storage of vegetables and other products post-harvest [6]. However, the application of nanotechnology in plant sciences has received little interest compared to nanomedicine and nanopharmacology [7]. The application of nanoparticles (NPs) in different crop plants has been evaluated, and the effects of these vary greatly with plant species and other factors as dose and type of NPs [8]. The use of NPs as nanofertilizers is common as they enhance nutrient use efficiency and increase yields through optimized nutrient management [7]. However, the use of NPs as biostimulants of biocompounds in plant crops is little known. In this sense, it has been shown that Cu nanoparticles (nCu) and their concentrations have a stimulatory effect that is related to the induction of antioxidant activity [9]. Also, the application of nCu in chitosan hydrogels was favorable to tomato growth and quality, increasing the catalase activity in the leaves and the lycopene content in the fruit [10]. Moreover, same authors found that application both nCu in chitosan hydrogels or only chitosan hydrogels decreased the concentration of Cu in plant tissue, additionally the fruit shows the lowest concentration of this mineral [10]. 

On the other hand, the management of such low concentrations may represent a problem when applied to plants, especially when nanoparticles are directed to the ground, because they can be inactivated or leached or because the amount is so small that it is difficult to handle. Chitosan has the ability to chelate minerals and other nutrients, making them more available to plants [11]. This is possible via the binding of metals through the functional groups (amino and hydroxyl) of chitosan [12]. Chitosan is a linear polymer formed by monomers of d-Glucosamine, a natural product derived mainly from the chitin of crustacean shells. Therefore, due to the characteristics of the bonding with chitosan-PVA metals, it can function as a vehicle for the application of NPs.

Although the effect of nCu is known for various crops [8], it is necessary to increase knowledge of fruit quality and post-harvest life. Thus, the objective of this work was to evaluate the effect of the application of Cu nanoparticles, introduced in chitosan-PVA hydrogels, on the growth of jalapeño pepper plants, the antioxidant content in fruits, and their post-harvest characteristics.

## 2. Results and Discussion

**Figure 1:**
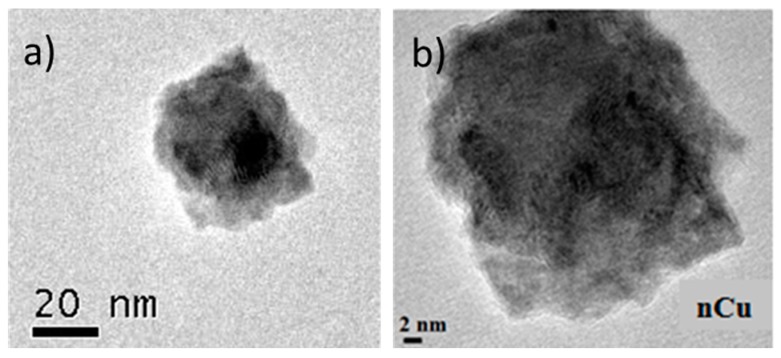
SEM (**a**) and TEM (**b**) images of nCu morphology.

**Figure 2:**
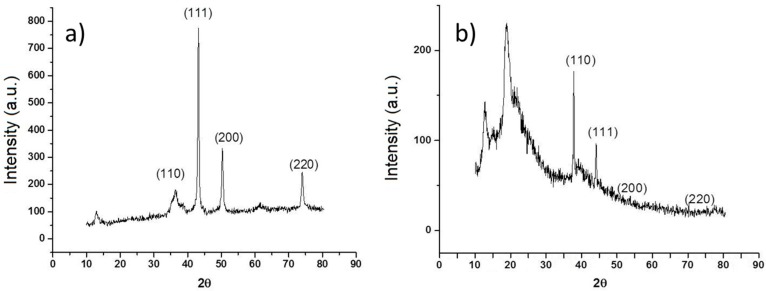
X-ray diffraction patterns of nCu (**a**) and nCu in hydrogels of chitosan-PVA (**b**).

Morphology of nCu was analyzed using SEM and TEM is shown in Figure 1a,b respectively. The sphere morphology is shown clearly in these figures. Also, Figure 2a shows the diffraction patterns of Cu nanoparticles, in their initial state, without any treatment, three important peaks were detected, reflections corresponding to angle 2θ: 43.6°, 50.8°, and 74.4° corresponding to the crystalline planes (111), (200), and (220) associated with diffraction patterns of elemental copper. In the same figure, reflections are observed at the 2θ angles of Cu_2_O at 29.9, 37, 42.6, 62.4, and 74.4 corresponding to the crystalline planes (110), (111), (200), (220), and (311) according to the Cu_2_O diffraction patterns obtained from the equipment database. The Figure 2b shows XRD pattern of the Cs-PVA hydrogel with Cu nanoparticles. This Figure shows a structure characterized by peaks at 2θ: 43.25° (111), 50.34° (200), and 74.04° (220), which match exactly with the standard data of Cu crystals. Also, it is possible to observe additional peaks at 2θ, that can be attributed to Cu_2_O, 37° (110), 42.6° (111), 62.4° (200), and 74.4° (220) and finally the peaks at 2θ between 10° and 20° are corresponding to amorphous polymer reflections.

The results of the growth and production variables of the jalapeño pepper plants are presented in Table 1. For the variable corresponding to the height of the plant, differences (*p* ≤ 0.05) were observed between the applied treatments. The highest height was observed in the absolute control, while the lowest height was observed in the Cs-PVA + 2.0 mg nCu treatment. Zuverza-Mena et al. [13] reported a decrease in growth in cilantro when applying 80 mg nCu kg^-1^ to soil. Additionally, they mentioned that this reduction in growth does not seem to be associated with assimilation and transport. However, Rizwan et al. [8] concluded that NP toxicity can be manifested in the plant by mechanisms such as genotoxicity, alterations in nutrient absorption, and generation of ROS. This can result in decreased plant growth.

Differences in the number of fruits per plant (*p* ≤ 0.05) were observed; the highest number of fruits was present in the Cs-PVA + 0.2 mg nCu treatment, while the lowest number was observed in the Cs-PVA + 2.0 mg nCu treatment (Table 1). This result is similar to Juárez-Maldonado et al. [10], who found an increase in the number of tomato fruits treated with 0.006 mg·L^−1^ nCu + chitosan. Moreover, differences (*p* ≤ 0.05) were observed in the average weight and total weight of fruits. The highest total weight of harvested fruits was obtained with the Cs-PVA and Cs-PVA + 0.2 mg nCu treatments, while the lowest total weight was observed for the Cs-PVA + 2.0 mg nCu treatment. For average fruit weight, the Cs-PVA treatment was best, and the lowest weight was observed in the Cs-PVA + 10 mg nCu treatment. This coincides with the results of a previous study [14] which demonstrates that exposure to NPs affects growth and development in several plant species. However, it differs from the report by Juárez-Maldonado et al. [10], who did not observe differences in the weight of tomato fruits treated with nCu + chitosan. It is common to observe this type of result with the application of nanoparticles in general, since both positive and negative effects on crop growth and yield have been reported [8]. For example, the application of nCuO reduces root development and outbreaks through the production of reactive oxygen species and lipid peroxidation. This is believed to occur through the interaction of NPs with proteins, membranes, nucleic acids, and metabolites, as well as free electrons on the surface of the NPs [15,16,17]. 

The dry and fresh weight of aerial biomass was not affected by the treatments since no differences were observed (*p* ≤ 0.05) (Table 1). Juárez-Maldonado et al. [10] observed a similar response in fresh aerial weight, as no differences were found in tomato plants treated with chitosan and nCu. The chitosan alone had no effect; this result differs from that found by Benavides-Mendoza et al. [18] who reported an increase in biomass in lettuce plants treated with this compound. This indicates that the response to the application of chitosan or nCu is different for each plant species; the soil characteristics and microbial composition could also affect these responses. In addition, it is known that the application of NPs can produce positive or toxic effects depending on dose, form, size, or plant species [8].

Table 2 presents the results of the variable corresponding to weight loss. Differences (*p* ≤ 0.05) were observed at 15 and 30 days of storage both at room temperature and under refrigeration. There was less weight loss in the control (5.80%) and the Cs-PVA + 2.0 mg nCu treatment (6.02%) when the fruits were refrigerated for 15 days. However, after 30 days of storage under the same condition, the Cs-PVA + 10 mg nCu treatment showed the lowest weight loss (20.52%). 

For fruits stored for 15 days at room temperature, the lowest recorded loss was observed in the treatment of Cs-PVA + 0.2 mg nCu (11.74%). At 30 days storage, the lowest weight loss was observed in the Cs-PVA + 0.02 mg nCu treatment (19%), and the highest weight loss was observed in the control fruits (20.79%). This behavior can be due to lignification of cell wall, since NPs can be translocated to the fruits [19], and it has been demonstrated that application of CuO NPs produce this effect on plants [20,21,22]. When the cell wall is lignificated, the fruit lost less water, as a consequence the fruits of the control present more weight loss that nCu treatments. 

The observed weight losses were higher than those reported by Hernández-Fuentes et al. [23] in bell pepper fruit var. California at 30 days storage under refrigeration conditions of 5 ± 1 °C (13.03%). However, this may be because of the lower storage temperature (5 °C) compared to this study. Báez-Sañudo et al. [24] reported that when fruits lose 6 to 7% of their weight, firmness and appearance decrease and consequently, quality declines. Espinosa-Torres et al. [25] found that for shelf conditions at 12 and 5 °C in Manzano pepper, the shelf life of fruits (one and two weeks, respectively) was prolonged. There was less weight loss and firmness in the fruits, without causing cold damage compared to ambient temperature (20 °C). This suggests that the shelf life of jalapeño peppers is approximately 15 days when stored under refrigeration (10 °C) and can be extended to lower temperatures, and values are lower when is maintained at room temperature. Therefore, the shelf life of jalapeño peppers can be extended with refrigeration due to decreasing metabolism as well as the degradation of polysaccharides [26].

The results of the total soluble solids (TSS) content are presented in Table 3. Differences (*p* ≤ 0.05) were observed at 0 and 15 days of storage at room temperature. The treatment of Cs-PVA + 2.0 mg nCu presented the highest TSS content, both at 0 days and at 15 days of storage, with values of 4.80 and 6.37 °Brix, respectively. In addition, a trend of increased TSS at up to 15 days of storage was generally observed. When fruits were refrigerated, differences (*p* ≤ 0.05) were observed at 0, 15, and 30 days of storage. In this case, unlike for fruits stored at room temperature, the TSS content remained more even. It was observed that the treatment of Cs-PVA + 2.0 mg nCu presented the highest values of TSS, especially at 30 days of storage (5.67 °Brix). However, there were differences between the values observed under refrigeration in the Cs-PVA + 2.0 mg nCu treatment compared to those obtained at room temperature, since in the latter condition there was 12% more TSS.

In general, an increase in the concentration of TSS was observed with the application of Cs-PVA + nCu; values up to 6.37 °Brix (Cs-PVA + 2.0 mg nCu) were obtained, exceeding those reported by Hernández-Fuentes et al. [23] in bell pepper fruits var. California stored under refrigeration at 5 ± 1 °C for 30 days (4.96 °Brix). This tendency to increase occurs because TSS increases as fruit matures, due to the degradation and biosynthesis of polysaccharides and the accumulation of simple sugars [26]. In addition, the accumulation of sugars in non-climacteric fruits is associated with the development of optimal quality for consumption [27]. Therefore, a higher accumulation of TSS represents higher quality fruit, as observed in this experiment when applying the Cs-PVA + nCu treatment. When the fruits are refrigerated, their metabolism decreases so the increase in TSS is not limited. In contrast to what was observed in this study, Juárez-Maldonado et al. [10] observed no difference in TSS in tomato fruits with the application of nCu or nCu + chitosan, indicating that the effect of the application of these types of compounds may be different for each vegetable.

For titratable acidity (TA), differences (*p* ≤ 0.05) were observed for almost all storage times under both room temperature and refrigeration. The results are presented in Table 4. In fruits stored at room temperature, a clear increasing tendency was observed as storage time passed in all treatments, including the control. In the case of fruits stored under refrigeration, this increasing tendency was observed only in the control. It has been reported that TA from different pepper cultivars increases with maturation. As the fruit matures, metabolic reactions increase the concentration of organic acids involved in the Krebs cycle [26]. Metabolism is reduced under refrigeration, resulting in lower TA content in the refrigerated fruits.

At the initial storage time, the treatment consisting of Cs-PVA + 2.0 mg nCu showed the highest value of titratable acidity in the fruits (0.65%). However, at 30 days of storage at ambient temperature, the Cs-PVA treatment exhibited the highest value (1.48% TA). In the case of refrigerated fruits at 30 days of storage, the control had the highest value (1.13% TA), surpassing the rest of the treatments. These acids serve as the energy reserve and participate in metabolic reactions for the synthesis of pigments, enzymes, and other materials and the degradation of pectins and celluloses that are essential for maturation processes [26]. The titratable acidity is therefore expected to increase with storage time.

Juárez-Maldonado et al. [10] reported values of 0.38% and 0.45% TA for tomato fruits treated with nCu + chitosan and chitosan, respectively. In the case of chitosan, the values were identical to those found here in jalapeño pepper (0.45%), whereas with the application of nCu, higher values were observed in jalapeño pepper compared to tomato. In addition, these authors reported an increase in the titratable acidity of tomato fruits treated with chitosan alone. This differs from what was found in the jalapeño pepper fruits in this study, since there were no differences between the control and the application of chitosan alone. Additionally, in the case of jalapeño peppers, the application of 0.2 and 2.0 mg nCu + Cs-PVA had a positive effect on the increase of TA. This suggests that both chitosan and nCu + Cs-PVA directly influence the behavior of this variable with storage time in pepper fruits.

The results regarding pH are presented in Table 5. At the initial evaluation time point, no differences (*p* ≤ 0.05) were observed between treatments, indicating that the application of Cs-PVA alone or with nCu does not affect this variable. However, at 15 days storage at both room temperature and refrigeration, the pH of all treatments was found to be statistically lower compared to the control. This differs from the report by Juárez-Maldonado et al. [10], since they recorded a pH increase in tomato fruits treated with chitosan + nCu, indicating that the effect may be different for each vegetable to which it is applied. At 30 days storage at room temperature, it was observed that treatment with Cs-PVA + 0.2 mg nCu had the lowest pH value (4.57), significantly different from the rest of the treatments. It was the only treatment in which pH decreased as storage time increased. In the case of 30 days of refrigeration, there was no significant difference between treatments. Hernández-Fuentes et al. [23] reported on bell pepper fruits var. California stored under refrigeration at 5 ± 1 °C; the pH values ranged from 5.98 to 6.08 at 0 and 30 days of storage, respectively, slightly higher than observed in this study. Tucker [28] reported that the pH in several fruits behaved inversely to variation in titratable acidity, whereas Hernández-Fuentes et al. [23] reported a similar tendency in bell pepper fruits, which coincides perfectly with that observed in the control jalapeño pepper fruits (Table 4 and Table 5). In treatments with Cs-PVA alone and with nCu, this trend is not observed, indicating that their application modifies pH behavior.

**Figure 3:**
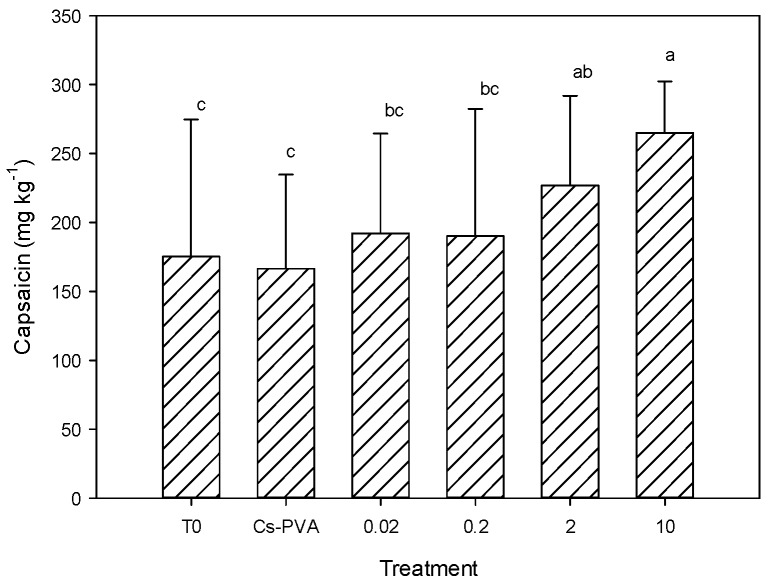
Capsaicin content in jalapeño pepper fruits. T0: Control; Cs-PVA: Only Chiosan-PVA; 0.02: Cs-PVA + 0.02 mg nCu; 0.2: Cs-PVA + 0.2 mg nCu; 2: Cs-PVA + 2.0 mg nCu; 10: Cs-PVA + 10 mg nCu. Means with the same letters within the same column are statistically the same according to the Fisher Least Significant Difference test (*p* ≤ 0.05). Bars represent standard deviation. Each datum is the average of five replicates.

Figure 3 shows the results of capsaicin content in jalapeno pepper fruits, where statistical differences between treatments were observed (*p* ≤ 0.05). It was observed that the Cs-PVA had no effect on this variable, since it presented values equal to the control. In contrast, the treatment of Cs-PVA + 10 mg nCu generated the highest concentration of capsaicin, exceeding the control by 51%. Additionally, the treatment of Cs-PVA + 2 mg nCu was greater than the control by 29%. In general, a clear trend of capsaicin increase was observed as nCu increased (Figure 3).

Capsaicin is one of the main antioxidants of jalapeno pepper [4] and protects cells from ROS. Therefore, it is possible that the observed increase in this compound is due to the induction of the antioxidant activity by the nCu [9], since these nanoparticles can interact with the intracellular structures [29], stimulating the formation of ROS. Ultimately, the plant defense system generates enzymatic and non-enzymatic antioxidant compounds [8], resulting in the accumulation of observed capsaicin (Figure 3).

The results of antioxidant capacity, total phenols and flavonoids are presented in Table 6. There are significant differences between treatments (*p* ≤ 0.05) in all variables, regardless of whether the pepper fruits were stored at room temperature or under refrigeration.

The antioxidant capacity of ABTS [2,2′-azino-bis (3-ethylbenzothiazolin-6-sulfonic acid)] in the fruits stored at room temperature was higher in the Cs-PVA + 0.02 mg nCu treatment [121.79 mg ascorbic acid equivalents (AAE) 100 g^−1^ Dry Weight (DW)] and 4% more than the control, while in the refrigerated fruits, the highest content was present in the control (120.22 mg AAE 100 g^−1^ DW).

The antioxidant capacity of DPPH (2,2-diphenyl-1-picrylhydrazyl) in fruits stored at room temperature was higher in the Cs-PVA + 10 mg nCu treatment (114.35 mg AAE 100 g^−1^ DW), followed by Cs-PVA + 2.0 mg nCu (109.90 mg AAE 100 g^−1^ DW), which were both higher than the control by 6.6% and 2.5%, respectively. In the refrigerated fruits, the highest value was obtained with Cs-PVA + 0.2 mg nCu (108.69 mg AAE 100 g^−1^ DW) followed by Cs-PVA + 10 mg nCu (103.74 mg AAE 100 g^−1^ DW). Both treatments were higher than the control by 23% and 18%, respectively, and lower than those obtained by Kim et al. [30] in the *Capsicum annum* Da-Bok cultivar (280.5 mg AAE 100 g^−1^ DW).

It is believed that the stimulatory effects of nCu are related to the induction of antioxidant activity [9], since they can interact with intracellular structures [29] by stimulating the formation of ROS, which in turn activates the antioxidant defense system of plants. This is possible since nanoparticles can cross cell walls [29] by several ways: endocytosis, pore formation, carrier proteins, or through plasmodesmata [19]. Even if there are the ion channels, they have size around 1 nm, thus nanoparticles are unlikely to cross the cell wall effectively [19]. The defense system of plants combines the generation of enzymatic and non-enzymatic antioxidant compounds [8], which can ultimately result in an increase of this type of compounds, as observed clearly in capsaicin content (Figure 3), and some treatments related to total phenols and flavonoids in both room temperature and refrigeration of this study (Table 6). The changes of enzymatic antioxidants have been demonstrated by Juárez-Maldonado et al. [10] in tomato plants treated with nCu + chitosan, where the catalase activity was more than five times higher than the control. This positive effect can be observed under light stress conditions by NPs, but can change under conditions of high stress where the activity of the antioxidant enzymes decreases due to the oxidative explosion [8]. Thus, it is possible to find different effects on antioxidant capacity depending on the dose of NPs used, as observed in the results of the present study (Table 6).

Total phenol content differences (*p* ≤ 0.05) were observed in fruits stored at both room temperature and under refrigeration. When the fruits were stored at room temperature, the Cs-PVA + 2.0 mg nCu treatment generated the highest value [64.71 mg galic acid equivalents (GAE) 100 g−1 DW], at 5.9% more than the control. On the other hand, in refrigerated fruits, the highest content was observed in the Cs-PVA + 0.2 mg nCu (63.18 mg GAE 100 g^−1^ DW) treatment, at 1.5% higher than the control. The values in both cases are much lower than those reported by Vega-Gálvez et al. [31] for *Capsicum annuum* L. var. Hungarian (1359 mg GAE 100 g^−1^ DW). However, in comparison to values reported by Juárez-Maldonado et al. [10] in tomato fruits treated with nCu + chitosan (5.8 mg GAE 100 g^−1^ DW), the values observed in jalapeño pepper were higher (approximately 11-fold). Ghasemnezhad et al. [26] reported 120 mg GAE 100 g^−1^ DW for the *Capsicum annuum* genotype Fox and 95 mg GAE 100 g^−1^ DW for the Arian genotype, higher than those found in the present study. Deepa et al. [32] also reported higher values for the pepper genotype Tanvi 186 mg GAE 100 g^−1^ DW and the genotype Flamingo 1122 mg 100 g^−1^ DW, as did Lee et al. [33] in the jalapeño var. Mitla (179.1 mg GAE 100 g^−1^ DW).

Flavonoids content differed (*p* ≤ 0.05) between fruits stored under ambient temperature and in refrigeration. In both cases, the best treatment was Cs-PVA + 0.02 mg nCu, surpassing the control by 13% and 17%, respectively. The observed response may be due to an induction effect of the antioxidant activity of nCu at low concentrations [9]. It was also observed that the highest flavonoid content was obtained for refrigerated fruits in almost all treatments. This is explained by the fact that the total flavonoid content decreases during maturation [34]. When the fruits are refrigerated, the maturation-related decrease is delayed so that the flavonoid content is maintained compared to fruits stored at room temperature. The highest flavonoid content was 277.29 mg equivalent of quercetin (EQ) 100 g^−1^ DW, in the Cs-PVA + 0.02 mg nCu treatment under ambient temperature conditions; this condition exhibited a content 10% higher than the control. Under refrigeration, 343.26 mg EQ 100 g^−1^ DW was observed with the same treatment, surpassing the control by 17%. These values are much higher than those reported by Ghasemnezhad et al. [26] in the *Capsicum annuum* genotypes Zorro (11.7 mg EQ 100 g^−1^ DW) and Arian (4.2 mg EQ 100 g^−1^ DW); Lee et al. [33] reported a lower value for the jalapeño var. Mitla (5.32 mg EQ 100 g^−1^ DW).

## 3. Materials and Methods 

### 3.1. Synthesis of Chitosan-Polyvinyl Alcohol (Cs-PVA) Hydrogels and Absorption of Cu Nanoparticles in Hydrogels

The synthesis of Cs-PVA hydrogels was performed in the pilot plant of the Applied Chemistry Research Center (CIQA). First, 250 mL of 2% chitosan (Marine Chemical and Mv = 200,000 g/mol) and 250 mL of 4% polyvinyl alcohol (PVA) (Aldrich, St. Louis, MO, USA, Mw = 30,000 to 50,000 and 98% hydrolysis) were dissolved by mixing for two hours at 300 rpm and 60 °C to obtain a hydrogel in a 1:2 ratio (Cs: PVA). Subsequently, 2.27 mL of the crosslinker (50% glutaraldehyde) was added at 450 rpm for 5 min at 25 °C. Then, 100 mL of 6% NaOH was added at 300 rpm and 25 °C for one hour. Finally, the Cs-PVA hydrogels were washed and purified with distilled water and ethanol, then dried and weighed. 

### 3.2. Characterization of Cu Nanoparticles

The Cu nanoparticles used in this work were acquired from Sky Spring Nanomaterials Inc. (Houston, TX, USA), and had a spherical morphology, 99.8% purity and an average diameter of 25 nm. The morphology of nCu was determined using a scanning electronic microscope (SEM) (JEOL-JMS-7401F) and Titan transmission microscope (TEM) (Model: JSM-7410). Also, Energy dispersive X-ray (EDX) analysis was done using Siemens EDX system, D5000 model.

### 3.3. Absorption of Cu Nanoparticles in Cs-PVA Hydrogels

To absorption of Cu nanoparticles in hydrogels first, 100 mg of the nCu were dispersed in a 1% Tween solution by ultrasound for 5 min (50 Watt power and 70% frequency), and then dilutions were prepared to obtain concentrations of 10, 2, 0.2, and 0.02 mg, which were subsequently absorbed each in 1 gram of Cs-PVA hydrogel and dried at a temperature of 60 °C.

### 3.4. Experimental Development

Jalapeño pepper plants (*Capsicum annuum* L.) hybrid var. Grande were established in a multi-tunnel greenhouse with a polyethylene cover at the Department of Horticulture of the Autonomous Agrarian University Antonio Narro and developed for 120 days after transplant (dat). The average temperature was 22.4 °C, while the active photosynthetic radiation was on average 677 μmol m^−2^ s^−1^ and the average relative humidity was 62%. Planting density was three plants per square meter. The substrate used was a mixture of peat moss and perlite (50:50, *v*/*v*), placed in black 12 L polyethylene bags. Before transplantation, 0.33 g of the solid Cs-PVA hydrogels were weighed for application of the treatments and distributed in the substrate in the lower, middle, and upper part of the pot, to cover a larger portion of the pot until 1 g of the hydrogel was added to each pot. A drip irrigation system was used to apply five irrigations per day, with an approximate application of 1.5 L per plant per day using a Steiner solution [35], with varying concentrations. The nutrient solution was applied to 25% during the vegetative growth of the crop, 50% during flowering and 75% during the development of the fruit [10]. The nutrient solution was maintained at pH 6.5. This nutrient solution (75%) contained 2.4 mg L^−1^ of Cu in a chelated form (EDTA). The treatments used were as follows: an absolute control, Cs-PVA, and four treatments with 0.02, 0.2, 2, and 10 mg (nCu) g^−1^ (Cs-PVA). These treatments were applied one time only prior to transplantation considering one gram of hydrogels per plant, resulting in a total of 0.02, 0.2, 2, and 10 mg of nCu per plant, respectively.

### 3.5. Variables for Growth and Production of Jalapeño Pepper

The number of fruits harvested per plant, the average weight of fruits (g), the total weight of harvested fruits and the fresh weight of aerial biomass (g) were registered to evaluate the growth and yield of the jalapeño pepper plants at the end of the crop. Dry weight of aerial biomass (g) was obtained after drying in a DHG9240A drying oven for 72 h at a constant temperature of 80 °C.

### 3.6. Storage of Jalapeño Pepper Fruits

The fruits were harvested at 90 days after planting and were selected for uniform color and to verify that they did not present physical and pathological damage. Once harvested, the fruits were divided into lots of 50 fruits per treatment; each fruit constituted an experimental unit. To evaluate the effect of nCu on post-harvest quality and behavior, jalapeño pepper fruits were stored at room temperature (20 ± 1 °C) and in cold storage conditions (10 ± 1 °C and 80% relative humidity), and three storage times (0, 15, and 30 days) were evaluated. There were six repetitions for fresh weight loss and three repetitions for physicochemical and functional analysis.

### 3.7. Physicochemical Analysis

The weight changes experienced by the fruits during the storage period were measured using a digital scale (OHAUS). Weight loss was reported as a percentage of accumulated losses with respect to the initial weight of the fruit.

For determination of the physicochemical variables, the fruits were cut into slices and ground in a knife mill (RTSCH GM 200, Haan, Germany). Total soluble solids (°Brix) were determined using a digital refractometer (PR-101, ATAGO PALETTE, Tokyo, Japan). The pH was measured using a digital potentiometer (Hanna Instruments, Woonsocket, RI, USA) and titratable acidity was determined using the AOAC method (942.15) based on titration of the sample with 0.1 M NaOH to pH 8.2 using phenolphthalein as an indicator [36]; titratable acidity was reported in % of citric acid on fresh weight.

### 3.8. Functional Analysis

The capsaicin content in fruits was determined by the method of Bennett and Kirby [37]. The ripe fruits were lyophilized and macerated in mortar. One gram of the sample was weighed, 10 mL of absolute ethanol was added and the mixture was stirred for 15 min. It was filtered on Whatman No. 1 and was gauged at 25 mL ethanol. The sample was transferred to a separatory funnel and 2.5 mL of buffer at pH 2.8, 0.5 mL of ethanol, 20.5 mL distilled water, and 10 mL of adogen-toluene solution were added. The mixture was vigorously stirred for 1 min. Subsequently, the absorbance of capsaicin was determined in the organic phase in a spectrophotometer (Varian CARY 100BIO, Turin, Italy) at a wavelength of 286 nm. To determine the capsaicin content in the samples, a calibration curve was constructed with this antioxidant (Sigma, Co, Kawasaki, Japan) in a range of 0 to 0.40 mg mL^−1^, dissolved in the solvents mentioned.

To prepare samples for the next functional analyses, the fruits were sliced and stored in an ultra-freezer at −70 °C (3003 Ultrafreezer Thermo Scientific, Waltham, MA, USA) for one week and lyophilized at 133 × 10^−3^ mbar (Labconco, FreeZone 6, Kansas City, MO, USA). Once lyophilized, the samples were ground in a knife mill (RTSCH GM 200, Haan, Germany) at 9000 rpm for 50 s until a fine powder of 150 μm diameter particles was obtained.

For the determination of the antioxidant activity of the ABTS [2,2′-azino-bis (3-ethylbenzothiazolin-6-sulfonic acid)] radical, the technique described in Re et al. [38] was employed, based on the discoloration of the radical ABTS cation. For sample preparation, 1 g of lyophilized powder was weighed and 20 mL of distilled water was added and centrifuged at 17,500 rpm for 10 min. Then, 1 mL of the supernatant was diluted in 20 mL of 80% methyl alcohol. The ABTS radical (7 μM) was reacted with potassium persulfate (Mallinckrodt Chemicals, St. Louis, MO, USA, 2.45 μM); the reagents were mixed in a ratio of 1:1. This mixture was left to stand, then covered for 16 h before starting the tests. Once the ABTS radical formed, it was diluted with 20% ethanol until it reached an absorbance of 0.7 ± 0.01 at 734 nm. The initial absorbance in the spectrophotometer (Varian CARY 100BIO, Turin, Italy) was measured in a quartz cell; then, 100 μL of pepper sample was added and stirred rapidly, and the change in absorbance 6 min into the reaction was recorded. Antioxidant activity was determined using a standard curve with ascorbic acid (95–125 mg AAE L^−1^). The results were expressed in milligrams of ascorbic acid equivalents per 100 g of dry weight (mg AAE 100 g^−1^ DW). 

To determine the antioxidant activity of the DPPH (2,2-diphenyl-1-picrylhydrazyl) radical, 1 g of lyophilized powder was weighed to prepare the sample and 20 mL of distilled water was added and centrifuged at 17,500 rpm for 10 min. Next, 1 mL of the supernatant was diluted in 20 mL of 80% methyl alcohol. Then, 2.5 mL of the DPPH (Sigma Aldrich, St. Louis, MO, USA) radical of a DPPH 6.1 × 10^−5^ M methanolic solution was placed in a quartz cell and reacted with 0.5 mL of the pepper sample. The mixture was allowed to stand in the dark for 30 min, and the change in absorbance was recorded at 517 nm in a spectrophotometer (Varian CARY 100BIO, Turin, Italy). The antioxidant activity was determined using a standard curve with ascorbic acid (0–80 mg L^−1^). The results were expressed as mg AAE 100 g^−1^ DW [39].

The determination of total phenols was carried out using the Folin–Ciocalteu methodology [40]. Briefly, 1 g of lyophilized pepper was weighed and hydrated with 20 mL of distilled water until a homogeneous mixture was obtained, and the suspension was then centrifuged at 17,500 rpm and decanted. Next, 0.5 mL of the supernatant was added to three test tubes, mixed with 2.5 mL of the diluted 0.2 N Folin–Ciocalteu reagent (1:10) (Sigma Aldrich, St. Louis, MO, USA) and allowed to stand for 5 min. Subsequently, 2 mL of a 7.5% sodium carbonate solution was added until a homogeneous mixture was achieved. This mixture was allowed to stand for 2 h, and the absorbance of the mixture was then read in a spectrophotometer (Varian CARY 100BIO, Turin, Italy) with quartz cells at a wavelength of 760 nm. The results obtained were expressed in milligrams of gallic acid equivalents per 100 g of dry weight (mg GAE 100 g^−1^ DW), according to the calibration curve for gallic acid and concentrations of 20–80 mg L^−1^. 

The total flavonoid content was determined using the Dowd method, adapted by Arvouet-Grand et al. [41]. A solution of 2% aluminum trichloride (AlCl_3_) (Fermont, Monterrey, Mexico) in methanol was used. First, 0.1 g of lyophilized pepper was weighed and suspended in 10 mL of methanol, then homogenized and filtered through paper (Whatman No. 1). Next, 2 mL of the filtered sample plus 2 mL of the AlCl_3_ methanolic solution was allowed to stand for 20 min in the dark. Subsequently, the assay was placed in a quartz cell, and the absorbance was recorded at a wavelength of 415 nm in a spectrophotometer (Varian CARY 100BIO, Turin, Italy). The total flavonoid content was determined using a calibration curve with quercetin for concentrations of 200–400 mg L^−1^. The results were expressed in milligrams equivalent of quercetin per 100 g of dry weight (mg EQ 100 g^−1^ DW).

### 3.9. Statistical Analysis

The crop was established using an experimental design of a Latin square (6 × 6) with 18 experimental units per treatment for growth and yield variables. A completely random design was used for the variables of physicochemical and functional analysis, with six and three experimental units respectively per treatment; only capsaicin has five experimental units. Statistical analysis of each variable was performed using the statistical program InfoStat (2016), in which an analysis of variance and a comparison of Fisher Least Significant Difference means (*p* ≤ 0.05) were performed.

## 4. Conclusions

The application of Cu nanoparticles in hydrogels of chitosan-PVA affected plant growth, especially at high concentrations, but increased the number and average weight of fruits. This indicates that the application of nCu generates both positive and negative effects on the growth and development of the jalapeño pepper crop, depending on the dose applied. Chitosan-PVA had no effect on jalapeño pepper plant.

The Cu nanoparticles in hydrogels of chitosan-PVA also minimized the weight loss of fruits stored for 30 days under both refrigeration and room temperature; they also increase the amount of total soluble solids in fruits stored at room temperature for 15 days. Cs-PVA shows more fruit weight loss at 15 days under refrigeration than the control, and had no effect to other conditions.

The application of Cu nanoparticles in hydrogels of chitosan-PVA increases the capsaicin content on pepper fruits. However, Cs-PVA alone had no effect on this variable.

In general, application of Cu nanoparticles in hydrogels of chitosan-PVA increased the content of antioxidants ABTS and DPPH, and the total phenols and flavonoids (4%, 6.6%, 5.9%, and 12.71%, respectively) in jalapeño pepper fruits stored for 15 days at room temperature. Refrigeration increased DPPH antioxidants, total phenols and flavonoids (23.9%, 1.54%, and 17.2%, respectively). Cs-PVA had no effect on these variables at room temperature. However, Cs-PVA decreases antioxidants ABTS and the total phenols under refrigeration (14.3 and 48.7, respectively); and the other hand antioxidants DPPH and flavonoids increase under this condition (9.7% and 2.6%, respectively).

The application of Cu nanoparticles in hydrogels of chitosan-PVA, even when applied to the substrate, not only had an effect on the development of the jalapeño pepper crop but also modified the post-harvest characteristics of jalapeño pepper fruits.

## Figures and Tables

**Table 1:** Plants of jalapeño peppers treated with Cs-PVA and different concentrations of nCu.

Treatment	Height (cm)	Number of Fruits	Average Fruits Weight (g)	Total Fruits Weight (g)	Fresh Weight Aerial Biomass (g)	Dry Weight Aerial Biomass (g)
Control	115.7 a	115.8 ab	26.6 ab	3086.8 ab	808.6 a	192.9 a
Cs-PVA	110.6 ab	124.5 ab	27.0 a	3450.3 a	848.0 a	187.8 a
Cs-PVA + 0.02 mg nCu	114.3 ab	115.6 ab	26.2 ab	3028.7 ab	766.6 a	171.0 a
Cs-PVA + 0.2 mg nCu	113.5 ab	126.6 a	26.4 ab	3342.2 a	812.1 a	185.2 a
Cs-PVA + 2.0 mg nCu	109.1 b	111.6 b	26.9 ab	3002.04 b	779.4 a	177.4 a
Cs-PVA + 10 mg nCu	112.3 ab	118.8 ab	25.3 b	3005.6 ab	797.3 a	182.1 a
CV (%)	7.53	18.84	9.14	17.13	17.81	20.16

Means with the same letters within the same column are statistically the same according to the Fisher Least Significant Difference test (*p* ≤ 0.05). CV (%): Coefficient of variation. Each data is the average of 18 replicates.

**Table 2:** Weight loss during storage time in Cs-PVA-treated jalapeño peppers and different concentrations stored at room temperature (20 ± 1 °C) and refrigeration (10 °C and 80% Relative Humidity).

Treatment	Refrigerated		Room Temperature	
	15 Days	30 Days	15 Days	30 Days
Control	5.80 c	23.06 a	12.66 ab	20.79 a
Cs-PVA	7.91 a	22.32 ab	13.76 a	20.15 ab
Cs-PVA + 0.02 mg nCu	7.84 ab	22.27 ab	12.80 ab	19.00 b
Cs-PVA + 0.2 mg nCu	7.30 abc	21.93 ab	11.74 b	20.53 a
Cs-PVA + 2.0 mg nCu	6.02 c	23.82 a	12.48 ab	20.31 a
Cs-PVA + 10 mg nCu	6.28 bc	20.52 b	13.31 a	20.26 ab
CV (%)	19.40	7.62	10.27	5.45

Means with the same letters within the same column are statistically the same according to the Fisher Least Significant Difference test (*p* ≤ 0.05). CV (%): Coefficient of variation. Each data is the average of six replicates.

**Table 3:** Behavior of total soluble solids (°Brix) during storage time in jalapeño pepper fruits treated with Cs-PVA and different concentrations of nCu stored at room temperature (20 ± 1 °C) and refrigeration (10 °C And 80% Relative Humidity).

Treatment	Room Temperature			Refrigerated		
	Initial	15 Days	30 Days	Initial	15 Days	30 Days
Control	4.47 ab	4.50 cd	5.30 a	4.47 ab	4.53 a	5.20 ab
Cs-PVA	4.33 ab	4.70 cd	5.17 a	4.33 ab	4.13 b	4.77 bc
Cs-PVA + 0.02 mg nCu	4.17 b	5.23 b	5.20 a	4.17 b	4.57 a	4.43 cd
Cs-PVA + 0.2 mg nCu	4.47 ab	4.37 d	5.50 a	4.47 ab	4.60 a	4.27 d
Cs-PVA + 2.0 mg nCu	4.80 a	6.37 a	4.83 a	4.80 a	4.47 a	5.67 a
Cs-PVA + 10 mg nCu	4.67 ab	4.80 c	4.67 a	4.67 ab	3.63 c	4.10 d
CV (%)	6.48	4.33	9.75	6.48	3.49	5.61

Values with the same letters within the same column are statistically the same according to the Fisher Least Significant Difference test (*p* ≤ 0.05). CV (%): Coefficient of variation. Each data is the average of six replicates.

**Table 4:** Behavior of titratable acidity (% citric acid) during storage time in jalapeño pepper fruits treated with Cs-PVA and different concentrations of nCu stored at room temperature (20 ± 1 °C) and refrigeration (10 °C and 80% Relative Humidity).

Treatment	Room Temperature			Refrigerated		
	Initial	15 Days	30 Days	Initial	15 Days	30 Days
Control	0.48 ab	0.61 a	1.24 abc	0.37 b	0.60 a	1.13 a
Cs-PVA	0.45 ab	0.85 a	1.48 a	0.45 ab	0.35 b	0.76 b
Cs-PVA + 0.02 mg nCu	0.35 b	0.72 a	1.01 c	0.35 b	0.61 a	0.74 b
Cs-PVA + 0.2 mg nCu	0.60 ab	0.73 a	1.36 ab	0.60 a	0.55 ab	0.87 b
Cs-PVA + 2.0 mg nCu	0.65 a	0.82 a	1.08 c	0.65 a	0.37 b	0.87 b
Cs-PVA + 10 mg nCu	0.37 b	0.74 a	1.11 bc	0.37 b	0.55 ab	0.74 b
CV (%)	24.63	19.76	12.56	5.61	25.05	23.92

Values with the same letters within the same column are statistically the same according to the Fisher Least Significant Difference test (*p* ≤ 0.05). CV (%): Coefficient of variation. Each datum is the average of six replicates.

**Table 5:** pH behavior during storage time in pepper fruits treated with Cs-PVA and different concentrations of nCu stored at room temperature (20 ± 1 °C) and refrigeration (10 °C and 80% Relative Humidity).

Treatment	Room Temperature			Refrigerated		
	Initial	15 Days	30 Days	Initial	15 Days	30 Days
Control	5.72 a	5.49 a	5.22 a	5.72 a	5.51 a	5.21 a
Cs-PVA	5.53 a	5.15 b	5.45 a	5.53 a	5.31 b	5.23 a
Cs-PVA + 0.02 mg nCu	5.50 a	5.08 bc	5.67 a	5.50 a	5.26 b	5.32 a
Cs-PVA + 0.2 mg nCu	5.72 a	5.16 b	4.57 b	5.72 a	5.21 b	5.32 a
Cs-PVA + 2.0 mg nCu	5.70 a	5.08 c	5.35 a	5.70 a	5.20 b	5.19 a
Cs-PVA + 10 mg nCu	5.88 a	5.17 b	5.46 a	5.88 a	5.26 b	5.30 a
CV (%)	4.52	1.23	4.77	4.52	1.62	1.52

Values with the same letters within the same column are statistically the same according to the Fisher Least Significant Difference test (*p* ≤ 0.05). CV (%): Coefficient of variation. Each data is the average of six replicates.

**Table 6:** Antioxidant capacity by ABTS [2,2′-azino-bis (3-ethylbenzothiazolin-6-sulfonic acid)] and DPPH(2,2-diphenyl-1-picrylhydrazyl), Total phenols and flavonoids in jalapeño pepper fruits treated with Cs-PVA and different concentrations of nCu stored at room temperature (20 ± 1 °C) and refrigeration (10 °C and 80% Relative Humidity) for 15 days.

Treatment	Room temperature				Refrigerated			
	ABTS	DPPH	TP	Fl	ABTS	DPPH	TP	Fl
Control	117.10 bc	107.27 c	61.07 b	245.92 d	120.22 a	87.68 d	62.22 b	292.85 c
Cs-PVA	115.94 cd	105.15 c	61.85 b	248.74 d	103.08 c	96.16 c	31.93 e	300.54 b
Cs-PVA + 0.02 mg nCu	121.79 a	78.03 e	54.29 c	277.20 a	102.27 c	108.69 a	56.44 d	343.23 a
Cs-PVA + 0.2 mg nCu	118.93 b	94.34 d	61.67 b	270.80 b	104.72 bc	82.93 e	63.18 a	262.59 e
Cs-PVA + 2.0 mg nCu	113.49 d	109.90 b	64.71 a	184.90 e	95.94 d	94.85 c	29.93 f	299.51 b
Cs-PVA + 10 mg nCu	114.10 d	114.35 a	55.44 c	261.31 c	108.66 b	103.74 b	59.93 c	276.95 d
CV (%)	1.25	1.25	1.32	0.66	2.19	1.63	1.01	0.55

ABTS (mg Ascorbic Acid Equivalents 100 g^−1^ Dry Weight); DPPH (mg Ascorbic Acid Equivalents 100 g^−1^ Dry Weight); TP: Total phenols (mg Galic Acid Equivalents 100 g^−1^ Dry Weight); Fl: Flavonoids (mg Equivalents of Quercetin 100 g^−1^ Dry Weight). Values with the same letters within the same column are statistically the same according to the Fisher Least Significant Difference test (*p* ≤ 0.05). CV (%): Coefficient of variation. Each data is the average of three replicates.
